# Exogenous ochronosis successfully treated with the combination of intense pulsed light and fractional CO_2_ laser^☆^

**DOI:** 10.1016/j.abd.2021.08.013

**Published:** 2022-10-20

**Authors:** William Wiegandt Ceglio, Mariana Figueiroa Careta, Regia Patriota, Luís Antônio Torezan

**Affiliations:** Department of Dermatology, Hospital das Clínicas, Faculty of Medicine, Universidade de São Paulo, São Paulo, SP, Brazil

Dear Editor,

A 52-year-old male patient, Fitzpatrick’s phototype V, using 4% hydroquinone on the face for two years due to a diagnosis of melasma, was referred to the Department of Dermatology at Hospital das Clínicas, Medical School, São Paulo complaining that, despite the treatment, the lesions had worsened. Dermatological examination showed confluent grayish macules on the malar prominence on both sides and brownish macules on the frontal, malar and nasal regions (Fig. 1).

**Figure 1:**
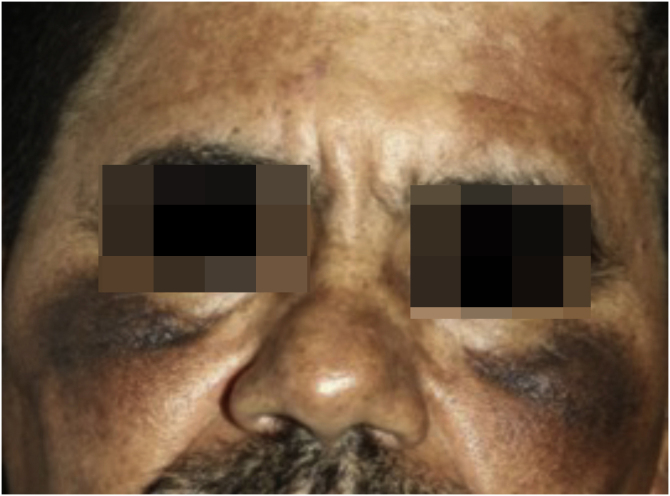
Patient showing grayish maculae on the malar prominences and brownish maculae on the frontal, malar and nasal regions.

Among diagnostic hypotheses, the possibility of exogenous ochronosis (EO) secondary to hydroquinone *versus* post-inflammatory hyperpigmentation was raised. An incisional biopsy was then performed, and histopathology detected a brownish-ochre pigment in the superficial dermis (Fig. 2), corroborating the suggestion of EO.

**Figure 2:**
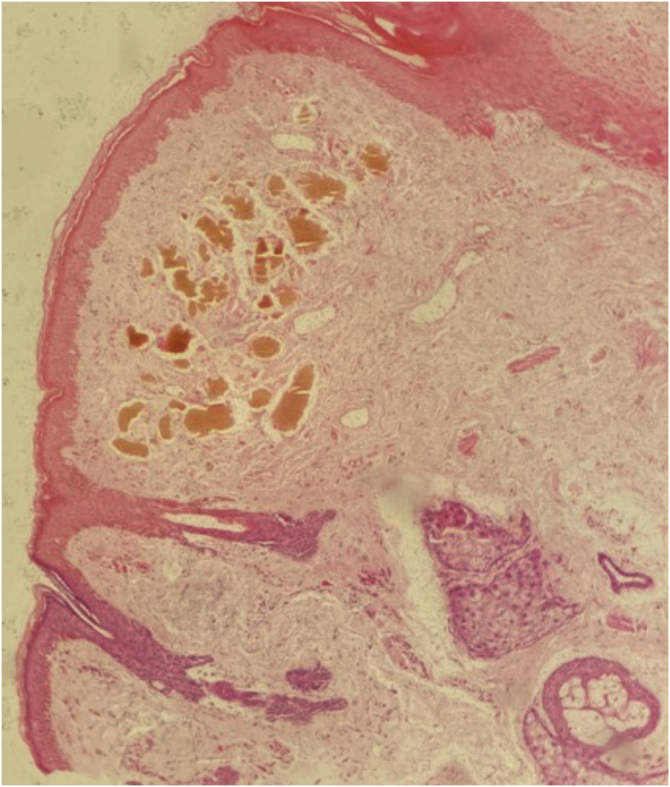
Histopathology of the skin of the left malar region revealing brownish-ocher pigmented clumps in the superficial reticular dermis and a mild interstitial and perivascular lymphohistiocytic infiltrate (Hematoxilin & eosin, 100X).

The patient was instructed to discontinue the use of hydroquinone and to use sunscreen. As therapy, the 1064 nm QS Nd:YAG laser (Etherea, Vydence Medical) was used, first during eight sessions per month (toning mode, fluence between 1.0‒1.3 J/cm^2^, spot size of 8 mm, frequency of 10 Hz), followed by four sessions a month with fluence between 4‒5 J and spot size of 5 mm, with slight improvement (Fig. 3). Subsequently, he underwent seven sessions, one per month, of intense pulsed light (IPL; Etherea, Vydence Medical; 580 nm filter, 15 J/cm^2^ fluence, 15 ms pulse), with partial response (Fig. 4). Finally, fractional 10,600 nm CO_2_ laser (Sculptor, Vydence Medical) was used with a 120 nm tip, energy between 80‒100 mJ and density of 50 MTZ/cm^2^ (total of five bimonthly sessions), with significant lesion improvement (Fig. 5). After each procedure, betamethasone 1 mg/g cream was prescribed daily for one week, in addition to recommendations to avoid sun exposure.

**Figure 3:**
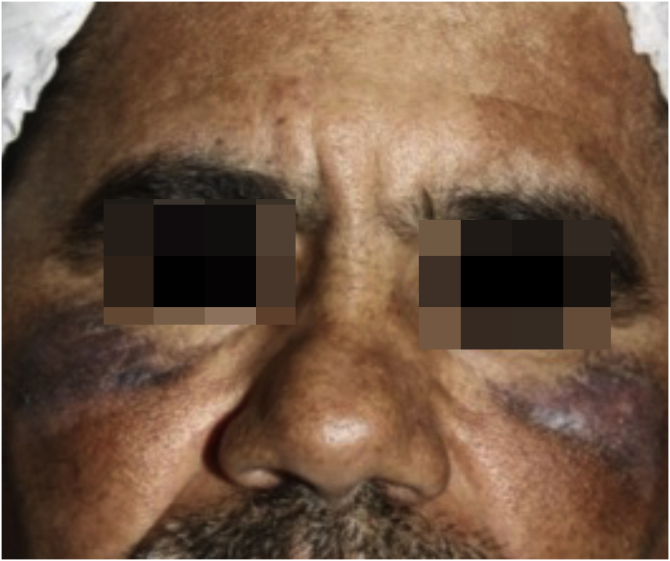
Slight improvement of lesions after the use of QS Nd:YAG 1064 nm laser (Etherea; Vydence Medical) for eight sessions in toning mode (fluence between 1.0‒1.3 J/cm^2^, 8-mm spot size and frequency of 10 Hz), followed by four sessions with a fluence of 4‒5 J and a spot size of 5 mm.

**Figure 4:**
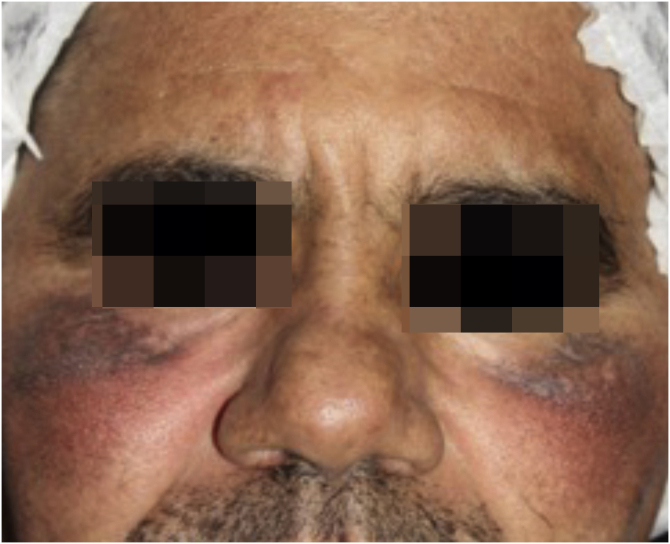
Partial response after seven sessions of Intense Pulsed Light (IPL; Etherea, Vydence Medical) using a 580 nm filter, with a fluence of 15 J/cm^2^ and a single pulse of 15 ms.

**Figure 5:**
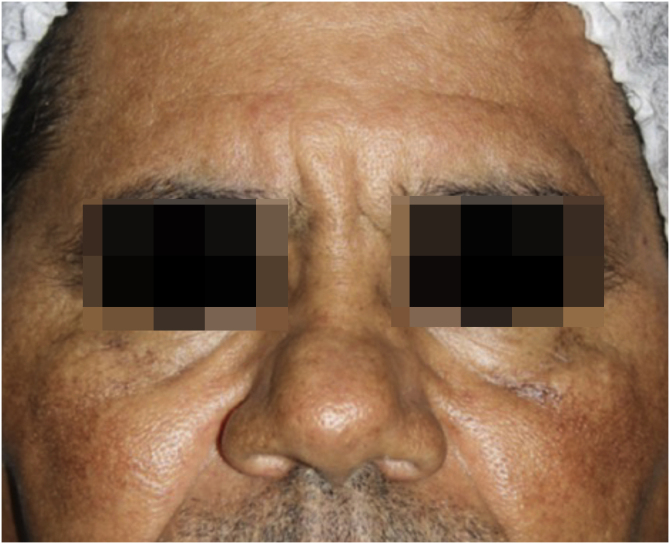
Significant lesion improvement after five sessions of fractional 10,600 nm CO_2_ laser (Sculptor, Vydence Medical) with 120 nm tip, energy between 80‒100 mJ and density of 50 MTZ/cm².

The term ochronosis was first used in 1866 by Virchow, related to the accumulation of an ocher-like brownish-yellow pigment on the histological examination of patients with endogenous ochronosis, a rare autosomal recessive disorder determined by a mutation that results in defective production of the homogentisic acid oxidase (HGO) enzyme with the accumulation of homogentisic acid (HG) in several tissues.^1^, ^2^, ^3^

Subsequently, the same patterns of skin pigmentation and histological findings were observed in patients using phenol, hydroquinone, resorcinol, mercury, and picric acid.^1^, ^2^, ^4^ In these cases, however, the lesions were restricted to the sites of exposure to the drug and there were no systemic alterations, thus receiving the name of EO.^2^, ^4^ Of the involved agents, hydroquinone is responsible for most of the cases and possibly results from the local inhibition of HGO activity, with subsequent accumulation of HG, which polymerizes to form the ochronotic pigment.^2^, ^5^

Clinically, EO presents as blue-gray macules or papules on photoexposed areas (mainly over bony prominences) of the face and cervical region.^2^ Histopathologically, short, curved, ocher-colored fibers (described as “banana bodies”) are identified in the papillary and superficial reticular dermis. In more advanced cases, due to the degeneration of the ochronotic fibers, the deposits appear amorphous and eosinophilic.^2^, ^3^, ^5^

As for treatment, discontinuation of the involved agent is recommended, but it can take years to see any improvement.^3^ Topical treatments are ineffective (trichloroacetic acid, cryotherapy) or little effective (retinoic acid, hydrocortisone).^2^, ^3^ Lasers are the most promising therapy, but they do not always show satisfactory results.^2^, ^3^ The first reported modality was the CO_2_ laser.^6^ Subsequently, Quality-Switched (QS) lasers started being used with satisfactory results, including Ruby (694 nm), Alexandrite (755 nm) and Nd:YAG (1,064 nm) lasers.^1^, ^4^, ^7^ More recently, improvement has been reported with the use of Intense Pulsed Light (IPL; 570 nm filter) and Nd:YAG picosecond laser (1064 nm), as well as laser combinations (QS Nd:YAG followed by CO_2_).^8^, ^9^, ^10^

For the present patient, the modality initially employed was the QS Nd:YAG laser, which acts in ochronosis possibly analogous to the removal of tattoo pigment from the dermis: pigment fragmentation and subsequent phagocytosis and elimination by lymphatic drainage or the transepidermal route.^7^ In the absence of a satisfactory answer, IPL is used; although the mechanism of lesion improvement is better understood in the treatment of epidermal lesions, the use of high energy and filters with higher cutoff points allows greater light penetration and the treatment of dermal lesions.^8^ It is noteworthy, however, that its use in patients with phototypes > IV should often be avoided or a test area is suggested, aiming at observing changes in pigmentation. In the present case, the application was safe and promoted a more significant improvement in relation to the described QS. Finally, the fractional CO_2_ laser was used, which possibly promotes extrusion of the pigment through the ablative microzones in the epidermis and dermis, helping to lighten the lesion.^10^

Therefore, the combination of different technologies, wavelengths and firing times resulted in an excellent response in the treatment of this patient, affected by a disease that always constitutes a therapeutic challenge.

## Financial support

None declared.

## Authors’ contributions

William Wiegandt Ceglio: Approval of the final version of the manuscript; drafting and editing of the manuscript; collection, analysis, and interpretation of data; critical review of the literature; critical review of the manuscript.

Mariana Figueiroa Careta: Approval of the final version of the manuscript; design and planning of the study; drafting and editing of the manuscript; collection, analysis, and interpretation of data; effective participation in research orientation; intellectual participation in the propaedeutic and/or therapeutic conduct of the studied cases; critical review of the literature; critical review of the manuscript.

Regia Patriota: Approval of the final version of the manuscript; design and planning of the study; effective participation in research orientation; intellectual participation in the propaedeutic and/or therapeutic conduct of the studied cases; critical review of the manuscript.

Luís Antônio Torezan: Approval of the final version of the manuscript; design and planning of the study; effective participation in research orientation; intellectual participation in the propaedeutic and/or therapeutic conduct of the studied cases; critical review of the manuscript.

## Conflicts of interest

None declared.
